# The Relevance of Complementary and Integrative Medicine in the COVID-19 Pandemic: A Qualitative Review of the Literature

**DOI:** 10.3389/fmed.2020.587749

**Published:** 2020-12-11

**Authors:** Georg Seifert, Michael Jeitler, Rainer Stange, Andreas Michalsen, Holger Cramer, Benno Brinkhaus, Tobias Esch, Annette Kerckhoff, Anna Paul, Michael Teut, Pirus Ghadjar, Jost Langhorst, Thomas Häupl, Vijay Murthy, Christian S. Kessler

**Affiliations:** ^1^Department of Paediatric Oncology/Haematology, Otto-Heubner Centre for Paediatric and Adolescent Medicine (OHC), Charité – Universitätsmedizin Berlin, Corporate Member of Freie Universität Berlin, Humboldt-Universität zu Berlin, and Berlin Institute of Health, Berlin, Germany; ^2^Department of Pediatrics, Faculty of Medicine, University of São Paulo, São Paulo, Brazil; ^3^Department of Internal and Integrative Medicine, Immanuel Krankenhaus Berlin, Berlin, Germany; ^4^Institute of Social Medicine, Epidemiology and Health Economics, Charité – Universitätsmedizin Berlin, Corporate Member of Freie Universität Berlin, Humboldt-Universität zu Berlin, and Berlin Institute of Health, Berlin, Germany; ^5^Department of Internal and Integrative Medicine, Evang. Kliniken Essen-Mitte, Faculty of Medicine, University of Duisburg-Essen, Essen, Germany; ^6^National Centre for Naturopathic Medicine, Southern Cross University, Lismore, NSW, Australia; ^7^Institute for Integrative Health Care and Health Promotion, University Clinic for Integrative Health Care, Faculty of Health, School of Medicine, Witten/Herdecke University, Witten, Germany; ^8^Department of Radiation Oncology, Charité – Universitätsmedizin Berlin, Corporate Member of Freie Universität Berlin, Humboldt-Universität zu Berlin, and Berlin Institute of Health, Berlin, Germany; ^9^Department of Internal and Integrative Medicine, Klinikum Bamberg, Chair for Integrative Medicine, University of Duisburg-Essen, Bamberg, Germany; ^10^Department of Rheumatology and Clinical Immunology, Charité – Universitätsmedizin Berlin, Corporate Member of Freie Universität Berlin, Humboldt-Universität zu Berlin, and Berlin Institute of Health, Berlin, Germany; ^11^Faculty of Medicine, Primary Care and Population Sciences, University of Southampton, Southampton, United Kingdom

**Keywords:** COVID-19, integrative medicine, complementary medicine, SARS-CoV-2, traditional medicine systems, phytomedicine, botanicals, mind-body medicine

## Abstract

**Background:** During the COVID-19 pandemic people are facing risks of adverse health effects due to the restrictions implemented such as quarantine measures, reduced social contact, and self-isolation. In this qualitative review, we collected data on potential preventive and therapeutic health benefits of Complementary and Integrative Medicine (CIM) that might be useful during the COVID-19 pandemic. We have reviewed the scientific literature to summarize CIM practices that could be beneficial for improving physical and mental health and well-being of the population under the current pandemic circumstances. It must be noted that this review is not SARS-CoV-2 specific and we explicitly do not intend to make any SARS-CoV-2 specific health claims in this article.

**Methods and Findings:** A qualitative, non-systematic literature review was conducted in Medline to identify literature describing preventive and therapeutic CIM approaches for strengthening mental and physical health. For a variety of CIM approaches clinical evidence was identified, indicating beneficial effects. CIM approaches include specific dietary measures and selected micronutrients, physical activity, techniques from Mind-Body Medicine, single botanicals or botanical compounds, and spending time in nature among others. The effects of CIM measures on conditions like obesity and hypertension are of special relevance here, as these conditions are considered as risk factors for a severe course of COVID-19. Moreover, a possibly direct effect of CIM approaches on immune functions and clinical parameters in respiratory tract infections, such as influenza, were identified. The findings of this review could be helpful for clinicians, patients, and the general population during the current pandemic when discussing and/or considering CIM options.

**Conclusions:** CIM offers a variety of preventive and therapeutic options for strengthening physical and mental resilience, which could also be useful in the current COVID-19 pandemic. The evidence of CIM approaches with a potential benefit in the COVID-19 pandemic in different areas is worth to be analyzed. While this qualitative review has several obvious limitations, it might serve as useful starting point for further research on this topic.

## Introduction

With the COVID-19 pandemic, mankind is facing a global health threat for which no specific therapy has yet been scientifically established. Worldwide, more than 46 million infections and 1.2 million deaths related to SARS-CoV-2 had occurred by the end of October 2020 (1). The COVID-19 pandemic as well as previous viral epi- and pandemic threats such as severe acute respiratory syndrome (SARS), Middle East respiratory syndrome (MERS), Ebola virus disease and swine flu warrant the assumption that such agents will continue to threaten health care systems, societies and economies worldwide (2, 3).

Individual lifestyles not only affect overall health and well-being, but can also affect related personal risk factors to the extent that they can contribute to the spread and negative consequences of communicable diseases (CD) (4, 5). Besides, nutrition and lifestyle of individuals and populations can potentially affect environmental sustainability and human health (6). While the main focus during a pandemic such as SARS-CoV-2 is on causal factors for the virus, both the physical and mental health of individuals and the population are also important, especially for those with risk factors (7).

The most commonly identified relevant risk factors for a severe course of the COVID-19 disease in intensive care unit (ICU) patients, are hypertension (48% non-survivors vs. 23% survivors), diabetes mellitus (31 vs. 14%), coronary heart disease (24 vs. 1%), chronic obstructive pulmonary disease (7 vs. 1%), and renal dysfunction (4 vs. 0%) (7). Here, CIM approaches may mitigate the aforementioned risk factors by fostering resilience, self-efficacy/self-empowerment, and other health-related resources of the individual facing the current health challenge (8).

Although lockdowns are an important safety measure to protect public health, several cross-sectional studies showed that they can lead to a variety of negative lifestyle changes including lack of exercise, “unhealthy” eating patterns, sleep disorders, and psychological symptoms including anxiety and depression (9, 10).

The fact that a vaccine against SARS-CoV-2 or a specific cure for COVID-19 is not yet available makes it necessary to explore how current preventive and therapeutic gaps could be bridged with *Complementary and Integrative Medicine* (CIM) interventions. Evidence-based CIM approaches, such as Mind-Body Medicine, nutritional medicine, phytomedicine, could complement, and personalize conventional medical strategies as part of overall health care management (11, 12).

CIM practices generally emphasize a holistic, patient-centered approach to health, healthcare, and well-being—often including psycho-emotional, functional, social, and even spiritual aspects (11, 12). There is a noteworthy body of CIM knowledge that has evolved over centuries, which has been used to prevent and manage a variety of diseases and is being increasingly used in health care systems worldwide (13)—also in so-called Traditional Medicine Systems (TMS). The World Health Organization (WHO) emphasizes the role of Traditional Medicine as a medical heritage “to promote universal health coverage by integrating traditional and complementary medicine services into health care service delivery and self-health care” (14). In international scientific handling, TMS are mainly subsumed under CIM.

The complex interrelationships between the immune system and a variety of lifestyle factors such as exercise, stress reduction, healthy nutrition, spending time in nature, positive inner attitudes, and well-being have already been demonstrated (15–20). In spite of the challenges faced by people across the globe due to restrictive lifestyle factors such as social distancing and quarantine measures, the general public and patients could utilize this time of forced domestic retreat of varying degrees to strengthen resilience through simple preventive means and self-care. Thus, CIM measures could be used to improve quality of life in an extraordinary situation such as the COVID-19 crisis that causes stress, fear, anxiety and depression amongst individuals and societies worldwide (8).

The findings of this review are not intended to serve as alternative recommendations to official public health measures or conventional medical advice. They should be understood as simple and effective, preferably evidence-based tools that may complement global disease control measures and mainstream treatment strategies. At the same time, there is a worldwide tendency even among national governments, to ignore and/or reject scientific data on coronavirus and COVID-19, combined with the proliferation of related unfounded and specific health claims. Therefore, the authors explicitly want to emphasize that the results presented in this article are not SARS-CoV-2-virus specific and that no COVID-19-specific CIM claims can be made based on the current evidence-base.

## Methods

A literature review in the electronic database “Medline” was carried out on July 24, 2020 and updated on October 29, 2020. Four authors (GS, MJ, AM, CK) have selected potential CIM interventions in a consensus process that could be useful in strengthening mental and physical health during the COVID-19 pandemic. Based on this the search strategy included the terms “complementary,” “integrative,” “traditional,” “medicine,” “CIM,” “CAM,” “stress,” “mind-body-medicine,” “meditation,” “yoga,” “qi gong,” “tai chi,” “phytomedicine,” “botanical medicine,” “positive wellbeing,” “optimism,” “resilience,” “virus,” “virus infection,” and “COVID-19” separately or combined by Boolean Operators. Relevant meta-analyses and original articles including prospective trials, epidemiological population-based studies and retrospective analysis, reviews and guidelines were included. Publications in English and German were included.

## Results

A large variety of studies, interventions, outcomes, studied sample sizes, treatment durations, and observation times were identified. According to the studies reviewed, CIM interventions appear to strengthen physical and mental health through: (1) nutrition and vitamin supplementation, (2) Mind-Body Medicine, (3) positive attitude of life and relationships, (4) exercise, (5) nature therapy, (6) aroma therapy, (7) sleep medicine, and (8) botanicals/phytomedicine. These topics are partly covered in so-called Traditional Medicine Systems (TMS). Relevant points are presented in detail below:

### Nutrition and Vitamin Supplementation

#### Nutrition

Nutrition is currently discussed not only as a game changer for planetary health (21) and the ongoing current climate crisis (22), but also as a potential source and reservoir for the emergence of viruses such as Ebola, MERS, SARS (23), and the development of multi-resistant bacteria (24) due to factory farming (25, 26) and wild animal markets (23). In contrast to the adverse effects of unhealthy food on health, specific diets, rich in antioxidants and anti-inflammatory nutrients, can have a significant impact with regard to disease prevention/management and longevity (27, 28).

Such an influence of nutrition on health is particularly important for older people, as eating habits that contain macro- and micronutrients as well as phytochemicals can have a positive influence on health (29). Above all nutrition can play a major role in the “individual susceptibility” to bacterial or viral infections and, if infected, in the course and outcome of the infectious disease.

Hypo- as well as hyper-caloric nutritional status, alongside certain micronutrient deficiencies have been identified as risk factors for susceptibility to infections (30). During the COVID-19 pandemic, older adults and patients with chronic diseases became particularly vulnerable and most at risk to nutrition imbalance (31). In the context of COVID-19, several position papers and suggestions for clinical investigation have been published; however, little observational data are available yet (31–34).

An optimized nutritional status can have a range of positive effects on the immune system (35). A predominantly plant-based diet, including e.g., fruits, vegetables, legumes, nuts, and olive oil, may have an influence on the susceptibility to infectious diseases; particularly foods containing potentially antimicrobial, antioxidant, anti-inflammatory, and immunomodulatory phytochemicals, such as bitter substances, vitamin C, mustard oils, herbs and spices, and herbal teas (19).

Nutrition, particularly when optimized for nutrient proportions, predominantly plant-based and organic (36), can exert relevant anti-inflammatory effects (37) and may have a long-term impact on general health and NCDs such as obesity, hypertension, and cancer (38, 39). There is growing evidence for negative correlations between plant-based nutrition, chronic inflammation (40), and pathological immune responses such as in bronchial asthma (41). A meta-analysis including 29 RCTs with 2.689 participants consumption of plant-based diets was associated with a reduction in the mean concentrations of CRP [effect size, −0.55 mg/l, 95% confidence intervals (CI): −0.78; −0.32, *I*^2^ = 94.4%] and IL-6 [effect size, −0.25 ng/l, 95% CI: −0.56; 0.06, *I*^2^ = 74%] (37).

NCDs predispose the individual to reduced immunity (40, 42), whereas enhanced nutrition protects from susceptibility to infectious agents; for example, a 5% higher proportional intake of fruit and vegetables is associated with a 12% lower hospitalization due to influenza infections (43), a lower risk of infection due to *Mycobacterium tuberculosis* exposure (44), and a lower frequency of urinary tract infections in pregnant women (45). Furthermore, the effectiveness of vaccination may be higher when the plant part of the diet increases (46).

In a recent systematic review, vitamins A and D showed a potential benefit in viral respiratory infections, especially in deficient populations (47). Among the trace elements, selenium and zinc have also shown beneficial immunomodulatory effects in viral respiratory infections (47). Although the nutritional status of patients with COVID-19 has not yet been adequately studied, there is preliminary evidence that nutrient-related disorders are associated with poorer disease progression and outbreak, probably associated with greater susceptibility to infection (48).

In the following, we would like to go into more detail about the vitamins D and C:

#### Vitamin D

Vitamin D3 has crucial influence on many functions of the immune system (49). In a randomized controlled trial (RCT) with supplementation of 400 IU/d, improvements of serum 25-hydroxyvitamin D concentrations were associated with at least a 1.5-fold alteration in the expression of 291 genes, many with relation to immune cell function and inflammatory control (50). Of potential clinical importance, vitamin D deficiency has been associated with increased prevalence of different viral conditions such as human immunodeficiency virus and hepatitis C. The role of vitamin D in influenza has remained controversial (51), although there are preventive studies that show lower incidences when supplements are given in schoolchildren (52). In this RCT Influenza A occurred in 18 of 167 (10.8%) children in the vitamin D group compared with 31 of 167 (18.6%) children in the placebo group [relative risk (RR), 0.58; 95% CI: 0.34, 0.99; *P* = 0.04]. A recent meta-analysis of 25 RCTs including 11.321 participants for vitamin D supplementation in respiratory tract infections, most of them assumed or proven to be viral, revealed a homogeneous risk reduction among all participants (adjusted odds ratio 0.88, 95% confidence interval 0.81–0.96; *p* for heterogeneity <0.001) (53).

Vitamin D3 deficiency has been shown to be a risk factor for the onset of acute respiratory distress syndrome (ARDS) (54). In the context of COVID-19 a recent retrospective population-based study in Israel showed an association between vitamin D deficiency and greater likelihood of both COVID-19 infection and hospitalization in 14,000 analyzed individuals (55). In contrast, another study analyzed data from 348.958 individuals in the United Kingdom and found no association between vitamin D levels and COVID-19 infection. Future (especially randomized) studies are warranted to clarify the effects of vitamin D supplementation in the context of COVID-19 infection.

A serum concentration of vitamin D3 (25-OH) of at least 20 ng/ml is considered to be sufficient for bone health. In a preventive setting for infectious diseases, an increased dose of 40–60 ng/mL (corr. 100–150 nmol/L) is recommended.

#### Vitamin C

In a systematic review of 31 RCTs with at least 0.2 g/d of orally administered vitamin C for the prevention of common colds, the incidence could not be reduced under normal conditions, some benefit was observed in the form of a reduction in the duration and/or severity of symptoms (56). However, five RCTs under conditions of unusual physical and/or thermal stress with above average performing participants demonstrated homogenous benefits with a pooled risk-ratio (RR) of 0.48 (56). This could be a model for long-term preventive use in reducing the incidence or severity of manifestation of COVID-19. Recently, a review concluded that oral vitamin C may reduce the duration of acute respiratory viral infections symptoms including fever, chest pain, chills and bodily aches and pains (57).

More recent clinical research on vitamin C has focused on higher doses, at times given intravenously, e.g., in intensive care medicine/intensive care unit (ICU). In a pooled analysis of 18 RCTs, length of ventilation, when necessary, and durations of overall stays in the ICU, mostly for cardiac reasons, were reduced (58). When shortening of ventilation was considered, eight RCTs with cardiac and septic indications gave a pooled reduction of 14% for all patients, or of 25% if ventilation was >10 h (59). In a RCT with patients with sepsis and ARDS, comparable to the situation with COVID-19 pneumonia, there was a difference in survival at day 28 of 17% in favor of vitamin C, but this was only a secondary parameter (60). On the other hand, a recently published RCT showed that treatment with intravenous vitamin C in intensive care patients with septic shock did not significantly improve the duration of time alive and free of vasopressor administration over 7 days (61). Ongoing RCTs are currently investigating the effects of intravenous high-dose vitamin C in the treatment of severe COVID-19 diseases [e.g., (62)].

### Mind-Body Medicine (MBM)

MBM is based on the assumption that interactions between the brain, mind, body, and behavior can be used to activate health-promoting pathways (63). It includes behavioral approaches and techniques in conjunction with exercise, relaxation, meditation, and stress-regulation interventions (63, 64). MBM has been shown to improve psychological parameters, reduce individual and cellular stress, inflammation, improve immune function, involving epigenetic pathways, thereby facilitating self- and autoregulation, and resilience in general (65–68).

The impact of psychological and behavioral parameters on health is particularly important in vulnerable phases of life such as childhood and old age (69) or in challenging situations (70).

Psychological stress for example can lead to an increased susceptibility to viral infections of the upper airways (71). The influence of chronic stress on health is enormous (66, 72). Both CIM and stress research make a clear distinction between the effects of chronic stress and acute—but moderate—stress on health (70). Even in positively perceived stress, the dose, and duration of such stress plays an important role, since even positively evaluated permanent stress may result in health risks (70).

Several CIM interventions could be useful in the field of stress reduction in a pandemic, e.g., using mindfulness, compassion, yoga, and meditation practices (15–17). The influence of psychological processes as well as psychological and behavioral interventions on the immune system (16, 66, 73, 74), positive psychological well-being (75), physiological functions (76, 77) as well as the mind's impact on chronic diseases is quite well-researched (78).

It is known that MBM therapies can have a positive influence on inflammatory activity and virus-specific immune responses (79). A finding that may be of particular interest in the context of the current pandemic is a possibly improved antibody response through meditation in persons with chronic stress, immunocompromised persons and older adults. Antibodies were measured directly in three studies (80–82), two of which examined the antibody response to influenza vaccination (82); one showed that mindfulness-based stress reduction (MBSR) resulted in a significantly greater increase in hemagglutination-inhibition influenza antibody titers (80). The second study showed no relevant changes in serum influenza antibodies or nasal Immunoglobulin A (IgA) in older adults. In a third study in older adults with administration of the keyhole limpet hemocyanin antigen, there was a relatively larger increase in Immunoglobulin G (IgG) immediately following a MBSR intervention (81).

Literature on meditation as a method for stress reduction shows relevant potential for application in the current pandemic (76, 77). A randomized study showed that MBSR may be a novel treatment approach for reducing social risk factors like loneliness as well as like molecular pro-inflammatory gene expression in older adults (83). In general, meditation seem to have a potential for inflammation reduction, including gene expression, cellular and chromosomal health (84). This also includes self-regulation and self-healing capacities, i.e., the innate restorative capacities of mind and body (64).

In addition, several other MBM interventions showed beneficial effects. Yoga has been shown to increase several parameters of the immune function (74), as well as reduce several COVID-19 associated risk factors, including hypertension (85), obesity (86), further cardiovascular risk factors (87) or chronic obstructive pulmonary disease. Yoga and meditation are discussed in this context—also from the perspective of Traditional Indian Medicine (TIM)—as a potentially effective tool in the context of the current pandemic because of its global popularity (88–90).

Also qigong is supported by a growing body of scientific evidence (91, 92). A meta-analysis on qigong demonstrates the effectiveness of qigong in improving cardiovascular risk factors in participants with metabolic syndrome (91).

Complex MBM-trainings that include relaxation, nutritional counseling, and exercise within the framework of a multimodality group program can positively influence cardiovascular risk factors for COVID-19 such as atherosclerosis and systolic blood pressure (93).

Further studies on CIM interventions in the field of MBM are warranted in the current pandemic, possibly taught via online courses.

### Positive Attitude to Life

Psychological stress has been found to lead to a significantly and dose-dependently higher infection rate with coronavirus type 229E and other seasonal pathogens (94). Emotional state may therefore be an important variable in the immune defense. A number of large epidemiological studies show the protective influence of a positive attitude toward life in chronic non-communicable diseases and further studies show that chronic stress is associated with detrimental outcomes in many health conditions (94). Positive psychological well-being does not only seem to be crucial for several cardiovascular issues, but for resilience in a broader sense (75). When life becomes meaningful and positive, it reduces the risk factors for chronic heart disease (75) and hypertension (95) by reducing high levels of stress hormones (96). A positive attitude toward life can have significant effects on survival in healthy and sick people (97). Furthermore, a positive attitude to life is associated with a lower risk of cardiovascular events and all-cause mortality (98).

### Relationships

Relationships are important variables for health and survival in a variety of diseases (99). Isolation and loneliness, feelings that are likely to result from quarantine (situations) (100), which may in turn exert significant negative influence on specific physiological parameters (73, 101) such as immune functions (102) and are equally detrimental to the health as that of smoking (103). Particularly in a situation of increased insecurity such as a pandemic, where social contacts have negative connotations, strengthening social relationships (100)—possibly also via outdoor and online services can be important. In general, maintain strong relationships may lead to better objective health status and perceptions of health (99).

### Exercise

The positive effects of exercise for general mental and physical health as well as for specific physiological functions including the immune system have been demonstrated in several systematic reviews and meta-analyses (18). Lack of exercise is one of the most common causes of chronic diseases, making patients more susceptible to infections and complicating disease courses (104, 105). Evidence on preventive effects of exercise for the elderly is overwhelmingly positive (106). CIM exercises offers a range of preventive possibilities that may also include MBM approaches (91, 92, 107–109). Exercise practiced in open air such as Nordic-walking or a walk in the forest may have greater overall positive effects than fitness training in a studio or indoor home situations (110, 111).

### Nature and Forest Therapy

Spending time in nature can be both a preventive and a therapeutic approach that makes use of targeted effects of natural stimuli in forests, urban green spaces, and therapeutic landscapes in order to promote health-related self-regulation mechanisms in individuals and communities (15, 112). Nature and forest therapy thus represent a simple, easily accessible, low-cost, sustainable, and effective supportive method for improving health parameters. Especially in the current pandemic nature therapy can be important, because it can be practiced in the open air and individually. Therefore, these measures will most likely play an important role in preventive medicine in the near future (113). Recent studies showed an overall stress-reducing effect on quality of life, well-being, functions of the autonomic nervous system, blood pressure, endocrine activity, happiness, mental health, immune activity, and even neighborhood satisfaction (114, 115). These are factors that could be valuable in the current pandemic.

### Aromatherapy

Closely related to nature and forest therapy is the field of aromatherapy. Preclinical and clinical research on the effects of certain bioactive compounds in essential oils, which are available in a large number of plants, has gained a boost and is increasingly published (116). Of particular interest are the effects of terpenes and terpenoids on the suppression of inflammatory and infection responses and the immunomodulatory properties of these compounds (117). Aromatherapy has been explicitly and/or implicitly part of CIM worldwide, particularly in the field of botanical medicine, where such properties are used to achieve specific therapeutic goals (118–120), for example to reduce anxiety (121). Due to their anti-microbial portfolio, several of these substances are of interest for the prevention of (respiratory) infections, the supportive treatment of conventional treatments, or a stand-alone CIM therapy in the case of a mild course of respiratory tract infections (122).

### Sleep

Healthy sleep is undoubtedly an essential resource for health. Sleep deficiency, poor sleep quality or shifts in the chronobiologic sleep rhythm are associated with an increase in chronic diseases (123). Insufficient sleep (<7 h) is also associated with a significant increase in upper respiratory tract infections (105, 124, 125). CIM offers a variety of ways to improve sleep duration and quality including the application of oil mixtures of aromatherapy (126), e.g., lavender, but also MBSR, yoga, and tai chi may improve sleep quality (127). A small study showed that progressive muscle relaxation might reduce anxiety and improve sleep quality in patients isolated with COVID-19 (128).

### Botanicals/Phytomedicine

The use of herbal substances for respiratory viral infections is widespread and there are clinical data that may be relevant during the current pandemic (8, 129). For the treatment of viral infections of the upper respiratory tract, there are many preclinical data available for individual components as well as for entire plant extracts. A selection of promising herbal medicines (Pelargonium root extract, *Sambucus nigra*, green Tea, Glycyrrhiza, Echinacea species, *Cistus incanus*, Cannabinoids) that may be relevant to the current COVID-19 pandemic are presented below. A recent meta-analysis including 7 RCTs, comprising a total of 732 adults, showed an advantage for Chinese herbal medicine (CHM) compared to standard care (SC) alone: CHM plus SC had a superior effect on the change of symptom and sign score (−1.30 by SMD, 95% CI [−2.43, −0.16]; 3 studies; *n* = 261, *P* = 0.03), on inflammatory marker C-reactive protein (CRP, mg/L; −11.82 by MD, 95% CI [−17.95, −5.69]; 5 studies; *n* = 325, *P* = 0.0002), on number of patients with improved lung CT scans (1.34 by risk ratio, 95% CI [1.19, 1.51]; 4 studies; *n* = 489, *P* < 0.00001) (130). No significant adverse events were recorded in the included RCTs. Several ongoing RCTs are investigating the effects of Chinese herbal medicine in the treatment of COVID-19.

#### Pelargonium Root Extract

In traditional South African medicine, the root Umckaloabo (from Pelargonium sidoides DC) has been the predominant medicine for airways infections. Use and research in Western countries have established its antibacterial and antiviral potential. When tested in several conventional assays against 14 types of viruses, it showed activity against e.g., parainfluenza virus, influenza A virus (strains H1N1 and H3N2), human coronavirus, but not against the avian influenza A virus (H5N1) (131). In another model the ciliary beat frequency increased as a factor of mucociliary clearance (132).

A systematic review of 8 RCTs using different extracts from roots of Pelargonium sidoides in a variety of upper respiratory tract infections showed moderate evidence in the treatment of acute rhinosinusitis, colds in adults and in acute bronchitis in children and adults (133). More recent reviews have confirmed this when they were limited to colds (meta-analysis of 5 RCTs) (134), and when they were limited to children and adolescents, but allowed for various respiratory infections (meta-analysis of 6 RCTs; narrative review of 8 RCTs) (135). The latter also concluded symptom-alleviating effects in special situations such as in asthmatic or immunocompromised children (136).

#### Sambucus Nigra

For centuries, TMS in Europe and North America have used black elderberry (*Sambucus nigra* L.) for colds and influenza. *In-vitro* activity against 18 strains of influenza was shown with a syrup (137), while infected chimpanzees had better recovery from influenza (138). In contrast to many other phytodrugs, *Sambucus nigra* was already tested in at least two placebo-controlled RCTs in times when no standard antiviral therapy was available. They differed by about one decade and locations (Israel and Norway, respectively), so different influenza strains were predominant. The results were very similar, as the relief of self-reported symptoms was accelerated by an average of 4 days. In both studies, syrup with a standardized content of flavonoids, which was considered to be the active ingredient, was administered (139, 140).

#### Green Tea

Catechins as a class of polyphenolic flavonoids are the main active ingredients of green tea as well as many other teas, which among other properties can strengthen the immunity against viral, especially influenza infections (141). The most prominent of these, epigallocatechin gallate (EGCG), even inhibited dengue virus infection *in vitro* regardless of the serotype and at concentrations reasonable for pharmacological application (142). Several epidemiological studies have suggested that regular consumption of green tea decreases influenza infection rates (143), while clinical trials have mostly used standardized catechin extracts. In one study, a cohort of low-risk health professionals took 1,000 mg/d of standardized green tea catechins or placebo over 150 days. The incidence of viral influenza was lowered by an odds ratio of 0.25, at the same time duration of symptoms was shorter (144).

In addition to optimizing systemic immune functions, experiments have been carried out to investigate the effects of gargling green tea to protect against the influenza virus at the point of first contact with the human mucosa in the throat. The pooled analysis of 5 RCTs gave a relative risk of 0.7 for the incidence of influenza for green tea gargling as compared with water gargling or no intervention (145).

#### Glycyrrhiza

Due to the lack of effective antiviral drugs, several phytodrugs have been used for antiviral indications, mostly in prevention and/or treatment of influenza and common cold (146). The saponin glycyrrhizin either from *Glycyrrhiza glabra* (Mediterranean world) or from *Glycyrrhiza uralensis* Fisher (Eastern Asia) is considered to be the active component of licorice roots used almost all over the world. Its immunomodulatory and anti-inflammatory effects are well-known. Mice infected with lethal doses of influenza virus had much better chances of survival after having been given glycyrrhizin (147). Uptake of influenza A virus into isolated human lung cells and its intracellular replication were clearly reduced, but only when the glycyrrhizin was given before infection (148). In spite of its frequently recommended use, it has been tested in RCTs only as one of four components of maoto, the most frequent formulation for influenza in the traditional Japanese medicine “Kampo.” According to a systematic review, it may decrease the duration of fever when used alone or in combination with standard neuroaminidase inhibitor therapies (149).

#### Echinacea Species

Numerous preparations of different parts and with different extracts of *Echinacea* species, esp. *purpurea* L. and *angustifolia* L. have been used for similar purposes as glycyrrhizin. A Cochrane review revealed small advantages in reducing incidence and course of common cold (150). A whole-plant hydroethanolic extract from freshly pressed Echinacea purpurea was given in an RCT to patients with proven influenza and was not inferior to standard therapy with oseltamivir (151). A recent review showed that study results were largely consistent with a decrease in pro-inflammatory cytokines that play a role in the progression of cytokine storm and ARDS. No studies are currently being conducted on the therapeutic effect of echinacea in the treatment of cytokine storm (152).

#### Cistus Incanus

In recent studies, promising anti-viral properties of the traditional Mediterranean medicinal plant *Cistus incanus* (or *Cistus creticus*) containing highly polymeric polyphenols as active ingredients were established in preclinical (153) and animal studies (154) in influenza models. However, clinical efficacy could only be shown in one RCT with common cold (155).

#### Cannabinoids

Recently, with reference to the “cytokine storm” associated to COVID-19, certain cannabinoids have also been discussed as potentially useful in this context, based on immunomodulatory properties associated to cannabinoids; however, the available evidence is exclusively from the preclinical area and does not allow any conclusions regarding potential effects of cannabinoids in the context of acute viral infections (156).

### Traditional Medicine Systems (TMS)

Many of the above presented aspects can be found as part of TMS. TMS have a vast and diverse heritage from across the world and the WHO defines TMS as an “essential resource in medical care” (14).

At present, the majority of Chinese COVID-19 patients are treated with Traditional Chinese Medicine (TCM) in addition to conventional medicine within the framework of official medical therapy strategies (157–159). Similar developments are currently evolving in India with Traditional Indian Medicine (TIM) as well (88, 90, 160).

The main mechanisms of action of TMS-based self-regulatory approaches are mainly attributed to changes in diet and lifestyle under preventive aspects (161). But TMS may also offer a variety of defined supportive treatment measures for infectious diseases, such as poly-herbal compound preparations (157, 158, 162). Their effects are usually much more unspecific than, for example, a targeted antiviral strategy tailored to individual virus subtypes. The non-specificity of TMS may indeed prove to be of advantage, for a broader universal applicability with fewer adverse effects.

There are a number of protocols for systematic reviews and meta-analyses in the field of TCM on various topics related to COVID-19 associated diseases, the results of which are to be published in the coming months (163–167). On the other hand TCM may include products from rare wildlife, thus TCM could act as a source of SARS-CoV-2 like novel viruses (168). In response to the rapid spread of SARS-CoV-2 and the assumed role of Chinese wildlife markets, the Chinese government issued a temporary ban on the hunting, trade, and consumption of numerous wildlife species in February 2020 (169). However, these measures affect only part of the wildlife trade: the government defined only certain wild animals as “special farm animals,” thus exempting them from the trade ban (170). The wildlife ban only applies to consumption, but not to use in TCM (169); this does not account for the largely vegetarian-oriented TMS of South Asia, for example Ayurveda (171).

### General Recommendations

**Figure 1:**
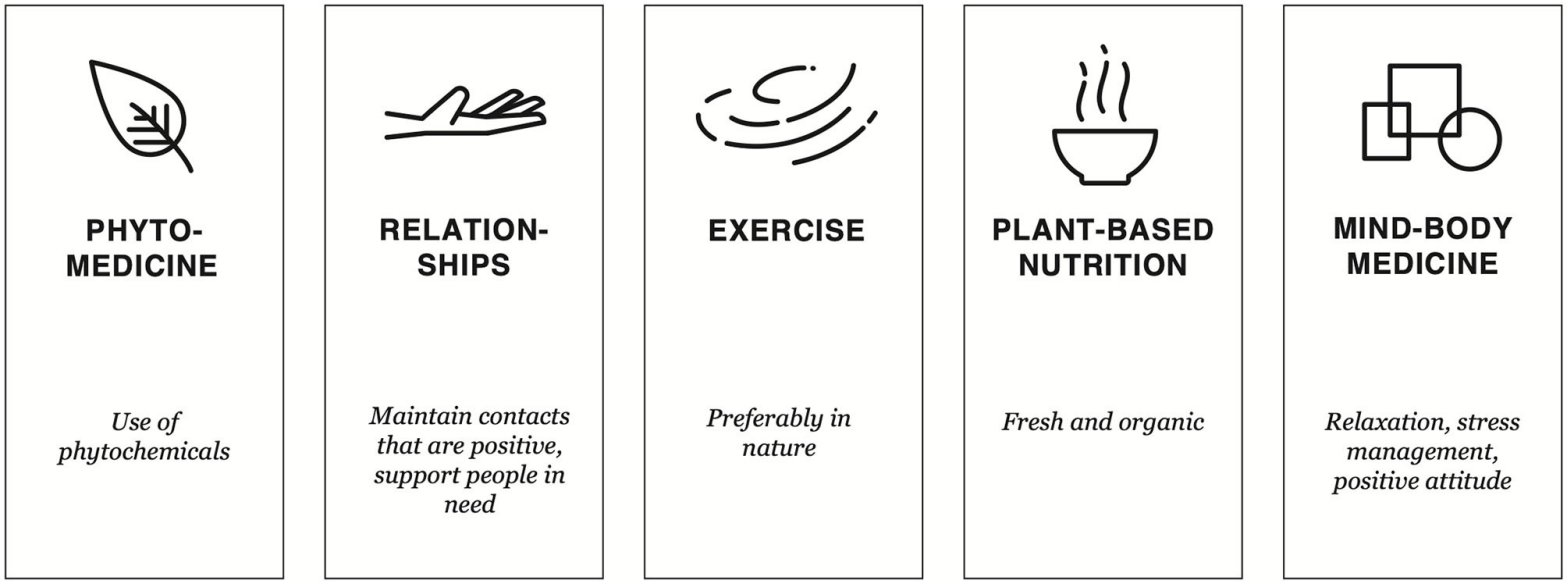
Key aspects of CIM in strengthening mental and physical health during a pandemic.

We have assembled a compendium of CIM recommendations formulated for clinicians, patients and the healthy population for general use, but these recommendations might be of particular benefit to people during the current COVID-19 pandemic (Figure 1 and Table 1).

**Table 1:** Recommendations for patients.

**Mind-Body Medicine and relaxation** Use the time available to encourage inner peace, contemplation, reflection, meditation (63). Maybe you can try out yoga, meditation, or other relaxation classes online.
**Strengthening relationships** **Affection**: Think of all those who are important in your life and what they mean to you in a positive sense. Exchange your thoughts with them and maintain communication (103). Make phone calls or online calls. Allow yourself to express your affection and gratitude. **Support others**: Call elderly people of your family and those who live on their own, entertain them by video calling, shop for others and just be there for them to ease the loneliness of the quarantine (172).
**Positive attitude** **Focus your attention on positive aspects** (94, 97): This lifts the spirits and strengthens resilience. Think about what you will do today that is beautiful and what positive things you want to do in the future. Limit the amount of time you spend reading or watching news about the current crisis. **Humor and laughter** (98): Even though the times are challenging, you can do things which makes you laugh or takes your mind off worrying. Read books which are funny, watch funny movies, swap jokes.
**Exercise** Do at least 15–30 min a day of morning stretches, walking, jogging or any other activity which suits you (18). Think creatively to use the available space at home such as, an open window, on the terrace, in the garden, a park, forest or wherever it is currently possible.
**Eat and drink healthy** (21) Try to eat regularly, preferably while being seated. Eat at least one warm main meal a day, preferably freshly cooked. Use a variety of kitchen herbs and spices. Drink plenty of water and incorporate warm drinks such as unsweetened herbal teas, ginger tea, teas with licorice, green tea into your diet. Drink small amounts of elderberry juice if available. • Chose fruits and vegetables with a range of colors. Eat plenty of fruit and vegetables, fresh or frozen, preferably organic. • Bitter food (e.g., constituents found in radicchio, endive, chicory, chard, eggplant, olives) • Food rich in vitamin C (e.g., found in parsley, peppers, cauliflower, turnip cabbage, broccoli, cress, lemon, oranges, Brussels sprouts, chives, sea buckthorn juice) • Mustard oils (e.g., found in garlic, leek, onion, spring onion, cabbage) • Herbs and spices (e.g., thyme, oregano, cloves, bay leaves, ginger, turmeric, basil, lemon balm, peppermint, rosemary, cinnamon—cook with them or prepare tea with them) • Integrate whole grain products and pulses. • If you prefer snacks/sweets foods, choose dark chocolate, nuts and seeds, dates, sweet fruit, licorice, cold extracted (forest) honey. • Reduce or avoid sugar and salt, highly glycemic foods and saturated fat (e.g., found in convenience food).
**Sleep well** Long and restful sleep strengthens the immune system. Practice a good sleep hygiene with regular sleeping hours by turning off screens and keeping the room cool, quiet and dark. If you feel cold, place a hot water bottle on your feet. Sleep for at least 7 h (127).
**Nutritional supplements** Maybe you don't need one. Most people can get all the vitamins and minerals they need through healthy eating. However, some people who do not get enough vitamins and minerals from food alone, or who suffer from certain diseases, might benefit from taking supplements, e.g., vitamin D (49).

*This list is only intended to help you identify lifestyle interventions that can strengthen your immune system. It is not intended to recommend any treatments, nor has any of them been shown to be effective against COVID-19. None of these practices are intended to replace other recommended treatments. Always consult your doctor or health care provider before starting*.

## Discussion

The aim of this qualitative review was to summarize the available evidence of CIM approaches with potential preventive and/or therapeutic relevance to the current COVID-19 pandemic. No SARS-CoV-2 specific claims are being made. Based on the available data, CIM could support coping strategies that could be helpful in dealing with the potential impact of public restriction measures imposed by the pandemic on the health and well-being of individuals and communities.

The relevance of CIM approaches in the COVID-19 pandemic is based mainly on the following four aspects: (1) CIM can have favorable effects on risk factors through lifestyle modifications such as diet, stress reduction, exercise, and other means of self-care (8, 158, 161), (2) CIM and especially MBM interventions can have a positive effect on stress and psychological parameters in a pandemic situation, particularly in the context of social isolation, anxiety, and depression (100), (3) CIM can strengthen the immune system in the case of non-communicable diseases (NCD) and CD (173), (4) CIM may have antimicrobial effects in case of targeted nutritional interventions and selected phytomedicine (8). The fact that the mentioned approaches and their mechanisms of action are not (yet) specific for SARS-CoV-2 and COVID-19 is not a convincing argument against the use of selected CIM measures in this global crisis. When few scientific data on effective and pathogen- and disease-specific preventive or therapeutic choices are available, considering CIM tools may be a wise response in our call for action during this pandemic.

Currently, no specific therapy for COVID-19 has shown to be effective that has been tested in at least one convincing RCT. Even for initially promising drugs, the evidence so far is negative (Hydroxychloroquine) or ambivalent (Remdesevir), while worldwide research in this field are developing with incredible urgency (174). In addition, proven preventive strategies are limited to an inhibition of any contact that facilitates virus transfer (175). Specific population groups at particular risk are the elderly, and patients suffering from COVID-19 with comorbidities such as chronic diseases like hypertension, diabetes, lung diseases, cancer, rheumatism, or autoimmune diseases. These population groups need strategies to cope with the current crisis (7). There is no proven or recommended strategy for people at risk to decrease their susceptibility, or if infected improve course and outcome (33). In addition, effective rehabilitation measures will be required for COVID-19 intensive care survivors. Here as well, it is important to identify specific and non-specific measures as well as their possible additive contributions from the fields like nutrition, physical exercise, phytomedicine, TMS, and MBM.

Another aim should be to improve immune functions through sufficient availability of essential micronutrients to build defense and physiological coping mechanisms, and to increase regenerative functions. CIM approaches may be a way to attempt delaying virus replication in the case of infection e.g., with the aid of selected foods for which antiviral effects have been demonstrated experimentally, though yet not for SARS-CoV-2, or which may indirectly improve the immune defense by reducing pathogens in other mucous membranes (such as the intestine) since there many food compounds with potential antiviral properties exist. Several different nutrients and vitamin compounds and applications have been found to improve and clean mucosal surfaces, thus interfering with viral replication on the mucosal surface and improving tissue homeostasis during viral replication and immune defense (132). For example, vitamin C is particularly important in the metabolism of immune cells and, as an antioxidant, is providing protection against collateral damage to cells and tissues caused by oxygen radicals as part of the immune defense. Regenerative processes in building and maintaining connective tissue structures are also improved by vitamin C, an effect that is gradually lost in scurvy, the vitamin C deficiency disease (176).

As previously discussed, many of the CIM measures may have the greatest effect in the field of prevention and strengthening of coping mechanisms as well as reduce risk factors for severe COVID-19 courses. However, CIM interventions could also help in the situation of acute infections. For example, many of the described substances are probably most effective when in direct contact with the pathogen in sufficient concentration already when consumed, such as when they can act as tea in the mouth and the throat area (e.g., gargling with green tea), when they act on the intestinal mucosa during digestion or when they are released back to respiratory surfaces from the circulation after absorption through the gut mucosa (e.g., garlic, ginger, thyme). A significant positive effect of these foods could also be their effect on the intestinal flora. The microbiome of the intestine is strongly influenced by our diet. Particularly with poor nutrition (rich in refined sugar, saturated fatty acids, animal protein, fried and processed foods), the burden by increased scavenging processes for macrophages and the stress to microbial metabolite removal for the immune system can weaken the defense capacity in acute infections. Therefore, a healthy, preferably plant-based diet could be of additional importance as a basic component of prevention (177–179).

Limitations of this non-systematic qualitative review include (1) presentation of CIM interventions and substances to the largest extend not (yet) tested on COVID-19 patients, (2) uncertainty about the safety of such interventions in case of COVID-19 infections, (3) uncertainty about which CIM intervention might be most appropriate for the individual patient, and (4) a non-systematic selection of CIM interventions. Thus, publication biases could not be excluded. In addition, most reported meta-analyses reported a very low to moderate quality of evidence. Following reviews and clinical studies should therefore systematically assess the safety of CIM interventions, conduct methodologically high-quality CIM studies in prevention and therapy of COVID-19 patient symptoms and systematically review present and further CIM interventions.

## Conclusion

During the current COVID-19 pandemic, robust evidence for effective prevention and specific therapy at the global level is not yet available. To date, there is no clinical evidence of a CIM measure to prevent or treat SARS-CoV-2-infection. Nevertheless, action must now be taken to reduce the potential negative impact of quarantine and physical distancing on human well-being and to reduce the morbidity and mortality of COVID-19 (180). CIM holds a substantial potential for building resilience and strengthening preventative resources through measures such as changing lifestyle and diet, using herbs, improving mental and physical health and reducing stress. In particular the fields of botanical medicine and aromatherapy should be subjects of further scientific attention and clinical research, as it is known from substantial preclinical and limited clinical research that many botanicals have properties that protect against respiratory viruses.

CIM offers a variety of easily feasible, accessible, evidence-based preventive, and therapeutic options for respiratory infections and for strengthening physical and mental resilience, likely to also help in prevention and treatment of COVID-19. An increased and sustaining use of the preventive and therapeutic potential of CIM in addition to the development of vaccination and specific treatment strategies for COVID-19 seems plausible and necessary. Although the amount of literature on CIM topics is steadily increasing, this does not necessarily correspond to an increase in high-quality evidence. Particularly, further clinical research is needed, including methodologically high-quality studies. Reviews should be continuously updated to provide a balanced view of the available data. In summary, more scientific work needs to be done to clarify the use of CIM interventions in the prevention and treatment of COVID-19. However, the implementation of potentially effective, safe and easily accessible CIM measures should already be considered now. In addition, there is a need to identify unsubstantiated claims of CIM measures with respect to SARS-CoV-2. All this would require a national or supranational initiative to facilitate the necessary substantial research to define possible contributions of CIM in the COVID-19 pandemic and beyond.

## Author Contributions

GS and CK conceived the manuscript, gathered information, and wrote part of the paper. MJ, RS, AM, HC, BB, TE, AK, AP, MT, PG, JL, TH, and VM gathered information and wrote part of the paper. All authors contributed to the article and approved the submitted version.

## Conflict of Interest

The authors declare that the research was conducted in the absence of any commercial or financial relationships that could be construed as a potential conflict of interest.
